# Multivariate analysis of oriental apple (*Malus orientalis* Uglitzk.) based on phenotypic and pomological characterizations

**DOI:** 10.1002/fsn3.2858

**Published:** 2022-03-29

**Authors:** Younes Moradi, Ali Khadivi, Farhad Mirheidari

**Affiliations:** ^1^ Department of Horticultural Sciences, Faculty of Agriculture and Natural Resources Arak University Arak Iran

**Keywords:** breeding, conservation, fruit, gene pool, *Malus orientalis*

## Abstract

Oriental apple (*Malus orientalis* Uglitzk.) is rich in valuable traits, such as later blooming, adaptation to a wider array of habitats, and capacity for longer storage of the apples, that should be considered important to the genetic makeup of the domestic apple. Here, the morphological diversity of this species was evaluated. There were significant differences among the accessions studied as revealed by the recorded traits. Ripening date ranged from late April to mid‐May. Fruit skin ground color showed strong diversity, including light cream, cream, yellow, light green, and green‐white. Also, fruit skin over color was highly variable, including white, cream, yellow, light green, and green‐white. Fruit weight ranged from 3.45 to 16.74 g. Principal component analysis (PCA) showed 13 PCs which contributed to 83.30% of total variance and fruit‐related characters were the most effective traits for separating and identifying the studied accessions. The Ward dendrogram reflected the similarities and dissimilarities among the accessions based on the qualitative and quantitative variables measured. A high phenotypic diversity within the collected material of *M. orientalis* was indicated. The diversity existing in the indigenous wild *M. orientalis* could further add new genetic information in the global gene pool of *Malus* species. The present study confirmed the necessity of preserving this unique genetic resource and continuing its study no matter the fact that in practice.

## Graphical abstract

A high phenotypic diversity within the wild collected material of *Malus orientalis* Uglitzk. was shown that could further add new genetic information in the global gene pool of *Malus* species. The present study confirmed the necessity of preserving this unique genetic resource and continuing its study no matter the fact that in practice.The diversity existing in the indigenous wild *M. orientalis* could further add new genetic information in the global gene pool of *Malus* species. The present study confirmed the necessity of preserving this unique genetic resource and continuing its study no matter the fact that in practice.

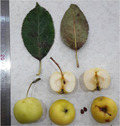

## 1 INTRODUCTION

Oriental apple (*Malus orientalis* Uglitzk.) is part of the species of the European group genus *Malus* (*Malus* Mill.), Order Rosales, family Rosaceae (Sokolov, 1954; Yuzepchuk, 1939). This is a medium‐sized tree (8–9 m) with a wide and hipped crown that blooms in April–May, with flowers 2.50–4.50 cm in diameter. The fruit is called pome, spherical, almost rounded, 20–30 mm in diameter with the remaining sepals. When the fruit ripens, the endocarpy becomes cartilaginous, and the outer tissues of the carpels become fleshy and completely merge with the tissues of hypanthium. On the cross section of the pericarp, the outer zone (epiderm and hypoderm—peel) and the parenchyma bordering on the dense cartilaginous tissue surrounding the nests of the ovary are clearly expressed. The main juicy part of the fruit (pome) is formed due to succulent tissue hypanthia. Fruit ripening occurs later (Kumachova, 2003; Levina, 1987).

According to recent molecular genetic studies, *M. orientalis* has played an important role in the formation of the *Malus domestica* Borkh. genome as a result of its spread along the Silk Road from the Central Asian region to the European (Cornille et al., 2012) region. According to Skibinskaya (1955), *M. orientalis* is the main ancestor of most European pubescent apple varieties.

*M. orientalis* was described by a lower diversity of fruit quality, but due to the high variability in populations, it could have contributed to the domestication of apple by introgression of some traits (Buttner, 2001). Other valuable traits, such as later blooming, adaptation to a wider array of habitats, and capacity for longer storage of the apples, should be considered important to the genetic makeup of the domestic apple (Forsline et al., 2003). Zhukovsky (1965) highlighted not only the late ripening and good transportability of fruits and high sugar content but also the low winter hardiness.

Collections of apple genetic resources are relatively long‐term investments that have been established for a variety of different purposes, including research, breeding, conservation, distribution, and public interest. National, subnational, or local gene banks, botanical gardens, arboreta, private companies, NGOs, and private individuals all maintain significant apple collections. Some apple gene banks have plantings of seedlings or grafted trees that represent wild species populations. These accessions offer the community access to flowers and fruits of wild species that can immediately be used in breeding programs or for phenotypic and genotypic evaluations (Bramel & Volk, 2019).

Morphological traits are the basic information for breeding programs, the management of genetic resources, the protection of cultivars, and the selection of candidates to diversify local production. Descriptors for morphological traits and identifying apple cultivars have been developed by the International Union for the Protection of New Varieties of Plants (UPOV, 2005). The objective of the present study was to assess the phenotypic diversity of *M. orientalis* accessions from three distinct populations in the Sistan‐va‐Baluchestan province from the southern part of Iran using quantitative and qualitative traits.

## 2 MATERIAL AND METHODS

### 2.1 Plant material

A total of 45 accessions of *M. orientalis* were sampled from three natural habitats of the Sistan‐va‐Baluchestan province from the southern part of Iran, including Maki, Rahmanabad, and Bonab. Maki area is located at 26°58'12"N latitude, 60°52'60"E longitude, and 932 m height above sea level. Rahmanabad area is located at 26˚58'30"N latitude, 60˚53'18"E longitude, and 943 m height above sea level. Bonab area is located at 26°44'35"N latitude, 61°06'45"E longitude, and 1133 m height above sea level. The appropriate distances were considered between the accessions in each collection site to avoid the possibility of sampling and collecting clones of the selected trees.

### 2.2 The characters evaluated

A total of 48 quantitative and qualitative morphological and pomological traits (Table 1) were used for phenotypic evaluations of the accessions selected. Fifty replicates per accession for leaf and fruit were used for measurements and the mean values were used for analysis. Fourteen characters, including leaf length, leaf width, petiole length, petiole width, fruit length, fruit width, fruit stalk length, fruit stalk diameter, endocarp diameter, mesocarp diameter, fruit flesh thickness, seed length, seed width, and seed thickness, were measured using a digital caliper (Model 10‐754‐500, Mitutoyo Cooperation). Fruit weight was measured using an electronic balance with 0.01 g precision (Model JTS‐JSA, Kia Cooperation). Also, the remaining characters were qualitatively estimated based on rating and coding according to the apple descriptor (UPOV, 2005; Table 2).

**TABLE 1:** Statistical descriptive parameters for morphological traits used to study *M. orientalis* accessions

No.	Character	Abbreviation	Unit	Min.	Max.	Mean	*SD*	CV (%)
1	Tree growth habit	TGH	Code	1	7	3.58	1.57	43.97
2	Tree growth vigor	TGV	Code	1	5	3.67	1.28	34.85
3	Branch skin color	BSC	Code	1	7	3.98	2.51	63.04
4	Tree height	THe	Code	1	5	3.44	1.27	36.95
5	Branching	B	Code	1	5	4.02	1.32	32.91
6	Branch density	BD	Code	1	5	4.20	1.16	27.62
7	Branch flexibility	BF	Code	1	5	3.00	1.28	42.63
8	Trunk type	TrTy	Code	1	5	2.73	1.51	55.46
9	Trunk diameter	TrDi	Code	1	5	3.22	1.55	48.14
10	Canopy density	CaD	Code	1	5	3.89	1.17	30.13
11	Tendency to form suckers	TeSu	Code	1	5	2.96	1.62	54.83
12	Leaf density	LD	Code	1	5	3.76	1.23	32.66
13	Leaf shape	LSh	Code	1	5	4.16	1.31	31.56
14	Leaf base shape	LBsSh	Code	1	3	2.33	0.95	40.90
15	Leaf length	LLe	mm	46.45	83.25	68.82	7.86	11.42
16	Leaf width	LWi	mm	25.73	46.69	35.77	4.98	13.91
17	Leaf shape index (L/W)	LShI	Ratio	1.50	2.49	1.94	0.24	12.23
18	Leaf area (L x W)	LA	mm^2^	1358.66	3815.51	2482.73	547.76	22.06
19	Leaf color	LC	Code	1	3	2.42	0.92	37.89
20	Leaf serration depth	LSeDp	Code	1	3	1.89	1.01	53.17
21	Petiole length	PeLe	mm	15.65	35.62	25.31	4.56	18.00
22	Petiole width	PeWi	mm	1.15	2.19	1.60	0.27	17.10
23	Petiole length/leaf length	PeLeWi	Ratio	0.26	0.48	0.37	0.06	15.73
24	Petiole color	PeC	Code	1	5	1.80	1.31	72.61
25	Ripening date	RiDa	Date	Late April	Mid‐May	2.69	1.41	52.45
26	Fruit yield	FrY	Code	1	5	3.76	1.23	32.66
27	Fruit length	FrLe	mm	15.35	29.50	24.73	3.78	15.30
28	Fruit width	FrWi	mm	20.40	36.16	29.83	3.96	13.28
29	Fruit weight	FrWe	g	3.45	16.74	10.68	3.54	33.13
30	Fruit shape	FrSh	Code	1	5	3.27	1.25	38.23
31	Fruit symmetry	FrSy	Code	0	1	0.22	0.42	190.91
32	Fruit apex shape	FrApSh	Code	1	3	1.09	0.42	38.26
33	Fruit stalk length	FrStLe	mm	14.13	39.57	23.30	7.35	31.55
34	Fruit stalk diameter	FrStDi	mm	1.14	1.75	1.48	0.17	11.46
35	Fruit skin ground color	FrSGC	Code	1	9	5.40	1.68	31.19
36	Fruit skin over color	FrSOC	Code	1	9	5.76	1.92	33.35
37	Color of spots on fruit skin	CSpFrS	Code	1	5	3.22	1.82	56.52
38	Endocarp diameter	EnDi	mm	3.01	12.64	7.39	2.01	27.23
39	Mesocarp diameter	MeDi	mm	6.95	16.97	12.48	2.59	20.76
40	Fruit flesh thickness	FrFlTh	mm	5.35	12.65	8.90	1.72	19.28
41	Fruit taste	FrTa	Code	1	7	3.49	1.14	32.69
42	Fruit quality	FrQ	Code	1	5	4.02	1.32	32.91
43	Seed presence	SePr	Code	1	3	1.27	0.69	54.17
44	Seed size	SeSi	Code	1	3	2.24	0.98	43.79
45	Seed number	SeNo	Number	1	5	2.04	0.95	46.67
46	Seed length	SeLe	mm	4.15	7.30	5.69	0.73	12.83
47	Seed width	SeWi	mm	2.63	4.36	3.52	0.47	13.43
48	Seed thickness	SeTh	mm	1.05	2.46	2.02	0.29	14.42

**TABLE 2:** Frequency distribution for the measured qualitative morphological characters in the studied *M. orientalis* accessions

Character	Frequency (no. of accessions)					
	0	1	3	5	7	9
Tree growth habit	‐	Weeping (6)	Spreading (23)	Open (13)	Semi‐erect (3)	‐
Tree growth vigor	‐	Low (4)	Moderate (22)	High (19)	‐	‐
Branch skin color	‐	Light‐brown (15)	Red‐brown (7)	Orange‐brown (9)	Brown (14)	‐
Tree height	‐	Low (5)	Moderate (25)	High (15)	‐	‐
Branching	‐	Low (4)	Moderate (14)	High (27)	‐	‐
Branch density	‐	Low (2)	Moderate (14)	High (29)	‐	‐
Branch flexibility	‐	Low (9)	Moderate (27)	High (9)	‐	‐
Trunk type	‐	Multi‐trunk/Low (16)	Multi‐trunk/Moderate (19)	Multi‐trunk/Low (10)	‐	‐
Trunk diameter	‐	Low (11)	Moderate (18)	High (16)	‐	‐
Canopy density	‐	Low (2)	Moderate (21)	High (22)	‐	‐
Tendency to form suckers	‐	Low (15)	Moderate (16)	High (14)	‐	‐
Leaf density	‐	Low (3)	Moderate (22)	High (20)	‐	‐
Leaf shape	‐	Oblong (4)	Ovate (11)	Lanceolate (30)	‐	‐
Leaf base shape	‐	Round (15)	Triangular (30)	‐	‐	‐
Leaf color	‐	Green (13)	Dark green (32)	‐	‐	‐
Leaf serration depth	‐	Low (25)	Moderate (20)	‐	‐	‐
Petiole color	‐	Light green (31)	Green (10)	Pink (4)	‐	‐
Ripening date	‐	Late April (15)	Early May (22)	Mid‐May (8)	‐	‐
Fruit yield	‐	Low (3)	Moderate (22)	High (20)	‐	‐
Fruit shape	‐	Flat (6)	Oblate (27)	Globular (12)	‐	‐
Fruit symmetry	Absent (35)	Present (10)	‐	‐	‐	‐
Fruit apex shape	‐	Flat (43)	Round (2)	‐	‐	‐
Fruit skin ground color	‐	Light cream (3)	Cream (2)	Yellow (24)	Light green (15)	Green‐white (1)
Fruit skin over color	‐	White (3)	Cream (5)	Yellow (10)	Light green (26)	Green‐white (1)
Color of spots on fruit skin	‐	White (16)	Yellow (8)	Green (21)	‐	‐
Fruit taste	‐	Sour (1)	Sour‐sweet (34)	Little‐sweet (8)	Sweet (2)	‐
Fruit quality	‐	Low (4)	Moderate (14)	High (27)	‐	‐
Seed presence	‐	Low (39)	Moderate (6)	‐	‐	‐
Seed size	‐	Small (17)	Medium (28)	‐	‐	‐

### 2.3 Statistical analysis

Analysis of variance (ANOVA) was performed to evaluate the variation among accessions based on the traits measured using SAS software (SAS Institute, 1990). Simple correlations between traits were determined using Pearson correlation coefficients (SPSS Inc., Norusis, 1998). Principal component analysis (PCA) was used to investigate the relationship between accessions and determine the main traits effective in accession segregation using SPSS software. Hierarchical cluster analysis (HCA) was performed using Ward's method and Euclidean coefficient using PAST software (Hammer et al., 2001). The first and second principal components (PC1/PC2) were used to create a scatter plot with PAST software.

## 3 RESULTS AND DISCUSSION

There were significant differences among the accessions studied as revealed by the recorded traits. Thirty‐five out of 48 characters measured showed a CV of more than 20.00%. The highest CV belonged to fruit symmetry (190.91%), in agreement with Khadivi et al. (2020) who observed these findings in *M. orientalis* from the Isfahan province, Iran. Also, the CV was more than 50.00% in petiole color (72.61%), branch skin color (63.04%), color of spots on fruit skin (56.52%), trunk type (55.46%), the tendency to form suckers (54.83%), seed presence (54.17%), leaf serration depth (53.17%), and ripening date (52.45%). The lowest CV belonged to leaf length (11.42%), fruit stalk diameter (11.46%), leaf shape index (12.23%), and seed length (12.83%) (Table 1). Five characters were the same and stable among the accessions, including leaf margin dentation (present), leaf serration shape (serrate), petiole cross‐section (round), fruit flesh color (white), and seed color (brown), in agreement with Khadivi et al. (2020) who observed these findings in *M. orientalis* from the Isfahan province, Iran.

Tree growth habit was predominantly spreading (23 accessions), while weeping (6), open (13), and semi‐erect (3) habits were also observed. Tree growth vigor, tree height, branch flexibility, trunk diameter, and leaf density were predominantly moderate. Branching and branch density were predominantly high. Canopy density was low (2 accessions), moderate (21), and high (22). Tendency to form suckers was low (15), moderate (16), and high (14). Leaf shape was predominantly lanceolate (30), while oblong (4) and ovate (11) shapes were also observed. Leaf base shape was round in 15 and triangular in 30 accessions. Leaf serration depth was low (25) and moderate (20). Petiole color was light green (31), green (10), and pink (4) (Table 2). The range of leaf length was 46.45–83.25 mm, leaf width was 25.73–46.69 mm, and leaf area was 1358.66–3815.51 mm^2^. Petiole length varied from 15.65 to 35.62 mm, and petiole width ranged from 1.15 to 2.19 mm (Table 1). Khadivi et al. (2020) reported the range of 38.56–101.36 mm for leaf length, 20.27–45.31 mm for leaf width, 899.05–4043.25 mm^2^ for leaf area, 12.77–46.30 mm for petiole length, and 0.45–2.68 mm for petiole width in *M. orientalis* accessions from the Isfahan province, Iran.

**FIGURE 1:**
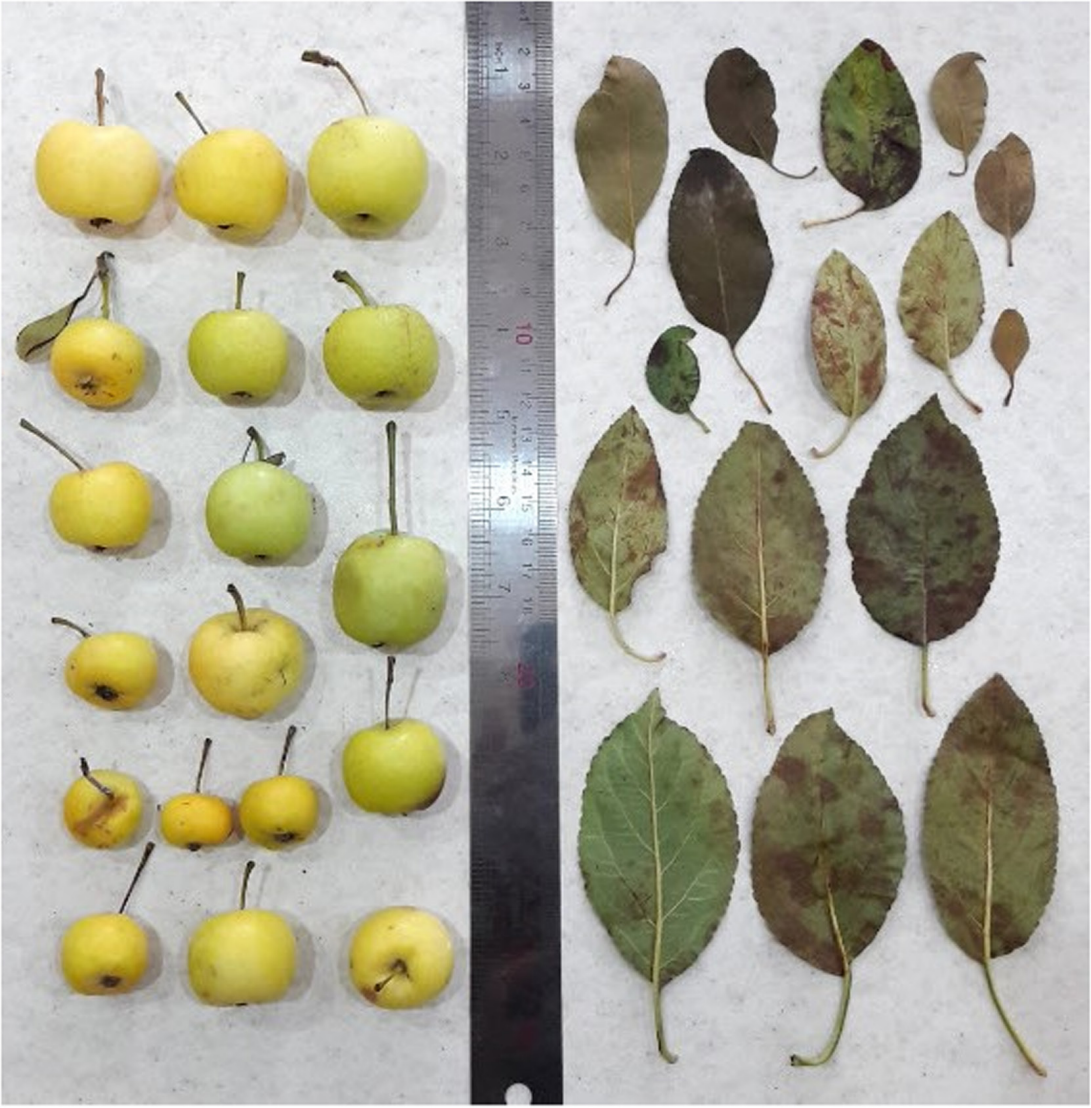
The pictures of leaves and fruits of *M. orientalis* accessions studied

Ripening date of 15 accessions was in late April, 22 accessions in early May (22), and 8 accessions in mid‐May. Fruit yield was low (3 accessions), moderate (22), and high (20). Oblate fruit shape was predominant (27 accessions), while flat (6) and globular (12) were also observed. Fruit apex shape was predominantly flat (43). Fruit symmetry was present only in 10 accessions, while fruit was asymmetric in 35 accessions. Fruit skin ground color showed strong diversity, including light cream (3 accessions), cream (2), yellow (24), light green (15), and green‐white (1). Also, fruit skin over color was highly variable, including white (3 accessions), cream (5), yellow (10), light green (26), and green‐white (1). Color of spots on fruit skin was white (16 accessions), yellow (8), and green (21). Fruit taste was predominantly sour‐sweet (34 accessions), and fruit quality was high in most of the accessions (27) (Table 2). The range of fruit‐related characters was as follows: fruit length, 15.35–29.50 mm; fruit width, 20.40–36.16 mm; and fruit weight, 3.45–16.74 g. Fruit stalk length ranged from 14.13 to 39.57 mm, and fruit stalk diameter varied between 1.14 and 1.75 mm. The range of endocarp diameter was 3.01–12.64 mm, mesocarp diameter was 6.95–16.97 mm, and fruit flesh thickness was 5.35–12.65 mm (Table 1). Khadivi et al. (2020) reported the range of 14.69–39.98 mm for fruit length, 22.16–47.39 mm for fruit width, 4.76–36.32 g for fruit weight, 9.10–39.40 mm for fruit stalk length, 0.87–2.50 mm for fruit stalk diameter, 6.74–17.18 mm for endocarp diameter, 12.20–29.70 mm for mesocarp diameter, and 4.08–11.51 mm for fruit flesh thickness in *M. orientalis* accessions from the Isfahan province, Iran. Hofer et al. (2013) reported an average of 27.07 mm for fruit length and 31.69 mm for fruit width in the *M. orientalis* accessions. Aldwinckle et al. (2002) recorded an average of 30.00 mm for fruit diameter in *M. orientalis* trees from the Russian Caucasus, while Ercisli et al. (2004) reported an average of 25.00 mm for fruit diameter in Turkish *M. orientalis*. The pictures of leaves and fruits of *M. orientalis* accessions studied are shown in Figure 1.

One to five seeds were observed per fruit and were low in 39 and moderate in six accessions. Seed size was medium in the majority of accessions (28). The range of seed‐related characters was as follows: seed length, 4.15–7.30 mm; seed width, 2.63–4.36 mm; and seed thickness, 1.05–2.46 mm. Khadivi et al. (2020) reported the range of 4.70–8.88 mm for seed length, 1.60–4.98 mm for seed width, and 0.96–3.18 mm for seed thickness in *M. orientalis* accessions from the Isfahan province, Iran.

Principal component analysis (PCA) was used to identify the patterns of variability among the accessions studied. For PCA, components with eigenvalues more than 1.00 were retained to uphold reliability of the final output. Thus, 13 PCs were observed which contributed 83.30% of total variance and the first three PCs explained 32.76% of the variance (Table 3). Khadivi et al. (2020) reported that the first 17 components accounted for 78.46% of total variance and the first three PCs explained 22.20% of total variance in *M. orientalis* accessions from the Isfahan province, Iran. The PC1 explained 15.83% of total variance and was represented by fruit length (0.93), fruit width (0.89), fruit weight (0.88), endocarp diameter (0.73), mesocarp diameter (0.78), seed size (0.73), seed length (0.68), and seed thickness (0.77) with positive correlations. The PC2 explained 9.26% of total variance and was constituted by branching (0.92), branch density (0.86), trunk diameter (0.56), canopy density (0.76), leaf density (0.69), and fruit yield (0.62) with positive correlations. The PC3 accounted for 7.68% of total variability and was represented by leaf shape (0.90), leaf base shape (0.93), leaf width (−0.72), leaf shape index (0.59), and leaf area (−0.56). These characters were the most effective traits for separating and identifying the studied accessions. The PCA has been widely used to analyze the phenotypic diversity of apple (Forsline, 2000; Hofer et al., 2013; Khadivi et al., 2020; Luby et al., 2001; Reig et al., 2015).

**TABLE 3:** Eigenvalues of the principal component axes from the PCA of the morphological characters in the studied *M. orientalis* accessions

	Component												
Character	1	2	3	4	5	6	7	8	9	10	11	12	13
Tree growth habit	0.11	−0.36	−0.04	0.02	−0.03	−0.09	0.77**	−0.05	−0.25	−0.07	0.09	0.06	0.09
Tree growth vigor	0.00	0.33	0.22	−0.02	0.06	−0.04	0.81**	−0.13	0.05	−0.01	0.08	0.00	−0.02
Branch skin color	−0.12	0.13	−0.01	−0.16	−0.08	−0.17	−0.07	0.08	0.03	0.14	−0.09	0.80**	−0.07
Tree height	−0.01	0.22	−0.20	0.14	0.14	−0.02	0.68**	0.02	−0.03	−0.13	0.38	−0.22	−0.08
Branching	−0.02	0.92**	−0.03	−0.01	−0.05	0.01	−0.14	0.12	0.10	0.00	−0.13	−0.03	−0.09
Branch density	−0.04	0.86**	0.10	0.10	−0.07	−0.06	0.01	0.24	0.12	−0.05	−0.14	−0.04	−0.16
Branch flexibility	−0.11	0.10	−0.16	−0.04	−0.07	0.08	−0.29	−0.10	0.06	0.00	−0.80**	0.05	0.06
Trunk type	−0.12	0.45	0.09	0.09	0.02	−0.10	−0.02	−0.08	0.71**	−0.06	−0.02	−0.01	0.06
Trunk diameter	−0.09	0.56**	−0.22	−0.12	−0.06	0.04	0.15	−0.34	−0.22	0.03	0.51	−0.03	0.16
Canopy density	0.05	0.76**	0.05	0.02	0.15	0.04	0.15	−0.09	−0.02	0.12	0.09	0.29	0.06
Tendency to form suckers	−0.02	0.02	0.08	−0.10	−0.13	−0.05	−0.13	0.02	0.86**	0.12	−0.07	0.02	−0.13
Leaf density	0.20	0.69**	0.02	−0.01	−0.36	0.13	−0.09	−0.17	0.32	0.05	0.07	0.13	0.09
Leaf shape	0.18	0.02	0.90**	0.01	0.06	−0.11	0.06	0.09	−0.03	0.12	0.06	0.13	−0.09
Leaf base shape	0.04	0.01	0.93**	0.00	0.01	−0.02	−0.03	0.10	−0.01	0.02	0.07	0.00	−0.12
Leaf length	0.36	−0.25	−0.23	0.52	0.48	−0.10	−0.02	0.21	0.10	−0.04	0.21	0.12	0.22
Leaf width	0.27	−0.19	−0.72**	0.13	0.14	−0.34	−0.03	0.29	−0.17	−0.10	0.10	0.19	−0.08
Leaf shape index (L/W)	0.03	−0.02	0.59**	0.35	0.29	0.33	−0.04	−0.12	0.29	0.11	0.06	−0.10	0.27
Leaf area (L x W)	0.36	−0.24	−0.56**	0.35	0.33	−0.26	−0.02	0.28	−0.05	−0.07	0.18	0.21	0.07
Leaf color	0.19	−0.01	0.09	0.01	0.83**	0.08	0.10	−0.05	−0.07	−0.13	0.04	−0.03	−0.17
Leaf serration depth	0.21	−0.12	−0.12	−0.02	−0.11	−0.23	0.00	0.04	−0.16	−0.24	0.04	−0.14	0.55**
Petiole length	−0.08	−0.01	−0.12	0.93**	0.13	0.11	0.06	0.07	−0.07	0.02	−0.04	0.00	0.10
Petiole width	0.19	−0.19	−0.25	0.10	0.64**	−0.10	−0.02	0.38	−0.05	0.26	−0.13	−0.01	0.31
Petiole length/leaf length	−0.41	0.21	0.05	0.67**	−0.21	0.23	0.09	−0.05	−0.18	0.07	−0.19	−0.03	−0.01
Petiole color	−0.10	−0.26	0.07	0.04	0.03	−0.12	−0.12	0.18	−0.42	0.27	−0.29	0.01	0.42
Ripening date	0.45	0.06	−0.12	0.20	0.34	0.17	0.40	0.25	0.10	−0.32	0.16	−0.01	−0.13
Fruit yield	−0.01	0.62**	0.16	−0.09	0.28	−0.02	0.35	−0.01	−0.05	−0.06	0.10	−0.21	−0.14
Fruit length	0.93**	0.02	0.03	0.13	0.21	0.02	0.05	−0.07	−0.12	0.10	0.00	−0.07	−0.01
Fruit width	0.89**	0.10	0.01	−0.01	0.23	0.03	−0.07	0.27	−0.10	0.01	−0.03	−0.04	0.06
Fruit weight	0.88**	0.07	−0.03	0.02	0.20	0.21	−0.02	0.26	−0.10	0.08	0.03	−0.03	0.07
Fruit shape	0.40	−0.09	−0.17	0.34	0.07	0.12	0.15	−0.65**	0.18	0.05	0.06	−0.10	−0.19
Fruit symmetry	−0.39	−0.33	−0.15	−0.03	−0.60**	−0.22	−0.07	−0.12	0.12	0.07	−0.12	0.19	−0.06
Fruit apex shape	0.15	−0.02	0.16	0.56**	0.04	0.10	−0.02	0.05	0.15	0.11	0.06	−0.18	−0.39
Fruit stalk length	0.54	0.01	0.19	0.56**	−0.07	0.14	−0.08	0.05	−0.04	0.21	0.09	−0.13	0.04
Fruit stalk diameter	0.11	−0.10	−0.26	0.19	0.29	0.44	−0.03	0.36	0.27	0.13	−0.22	0.05	0.49
Fruit skin ground color	−0.13	0.05	−0.20	0.36	0.04	0.02	−0.32	−0.14	−0.10	0.66**	0.28	−0.07	−0.18
Fruit skin over color	0.29	0.17	0.15	−0.09	−0.11	0.03	−0.09	0.43	0.10	0.65**	−0.15	0.19	0.02
Color of spots on fruit skin	0.22	−0.04	0.33	0.04	0.04	0.17	0.06	−0.07	0.21	0.75**	−0.01	−0.02	0.04
Endocarp diameter	0.73**	−0.10	0.01	0.10	−0.05	0.25	0.10	−0.16	−0.09	−0.02	−0.05	0.38	0.19
Mesocarp diameter	0.78**	−0.07	0.05	−0.05	−0.08	0.07	−0.10	−0.44	−0.05	0.13	−0.24	0.08	0.06
Fruit flesh thickness	0.28	0.07	−0.07	0.32	0.22	0.18	−0.04	0.80**	−0.03	−0.05	0.13	−0.01	0.02
Fruit taste	−0.25	0.05	−0.04	−0.14	0.23	0.26	0.04	0.12	0.17	−0.59**	0.05	−0.34	0.13
Fruit quality	0.42	−0.16	−0.15	−0.13	−0.06	0.31	0.42	0.03	0.39	0.13	−0.15	0.08	−0.12
Seed presence	0.16	0.09	0.02	0.19	−0.03	0.79**	−0.21	−0.06	−0.03	0.00	−0.05	−0.02	−0.07
Seed size	0.73**	−0.06	0.03	−0.16	0.07	0.13	−0.01	0.03	0.24	0.06	0.48	−0.08	−0.14
Seed number	0.29	−0.04	0.10	0.17	0.09	0.81**	0.11	0.09	−0.08	−0.01	−0.01	−0.12	−0.15
Seed length	0.68**	−0.15	−0.14	0.01	0.00	0.16	0.11	0.06	0.30	−0.07	0.19	0.35	0.09
Seed width	0.52	−0.02	0.17	−0.16	0.31	0.51	0.14	0.19	−0.03	0.12	0.27	−0.19	0.17
Seed thickness	0.77**	0.12	0.05	−0.09	0.02	−0.09	0.21	−0.01	0.12	0.14	0.04	−0.29	−0.13
Total	7.60	4.44	3.69	3.19	2.85	2.79	2.70	2.60	2.48	2.40	2.01	1.67	1.57
% of Variance	15.83	9.26	7.68	6.65	5.95	5.82	5.63	5.41	5.17	5.00	4.20	3.47	3.26
Cumulative %	15.83	25.09	32.76	39.41	45.36	51.17	56.80	62.21	67.38	72.38	76.57	80.04	83.30

Note

**Eigenvalues ≥0.55 are significant at the *p* ≤ .01 level.

**FIGURE 2:**
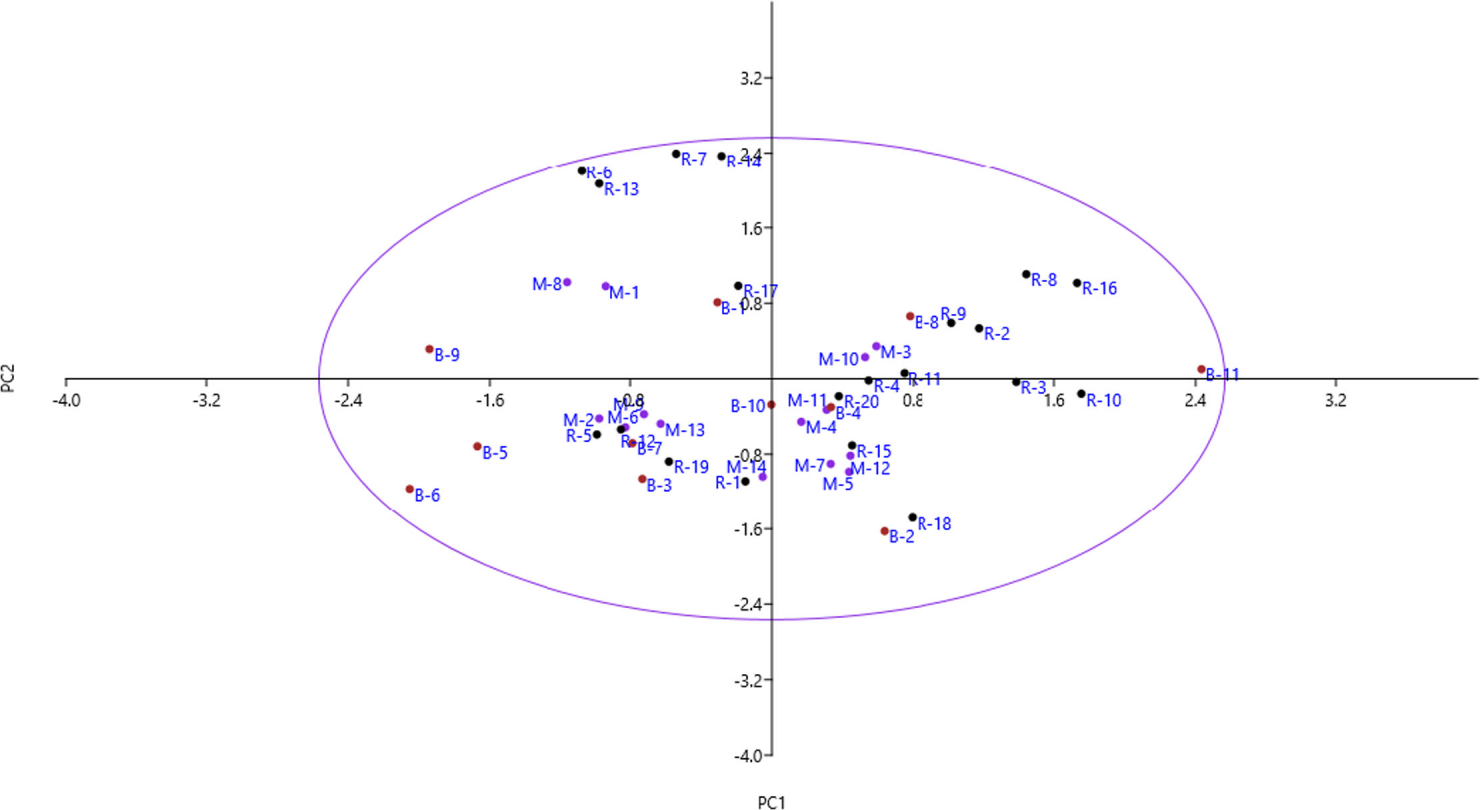
Scatter plot for the studied *M. orientalis* accessions based on PC1/PC2. The symbols represent the accessions of each area in the plot, including Maki (M), Rahmanabad (R), and Bonab (B)

**FIGURE 3:**
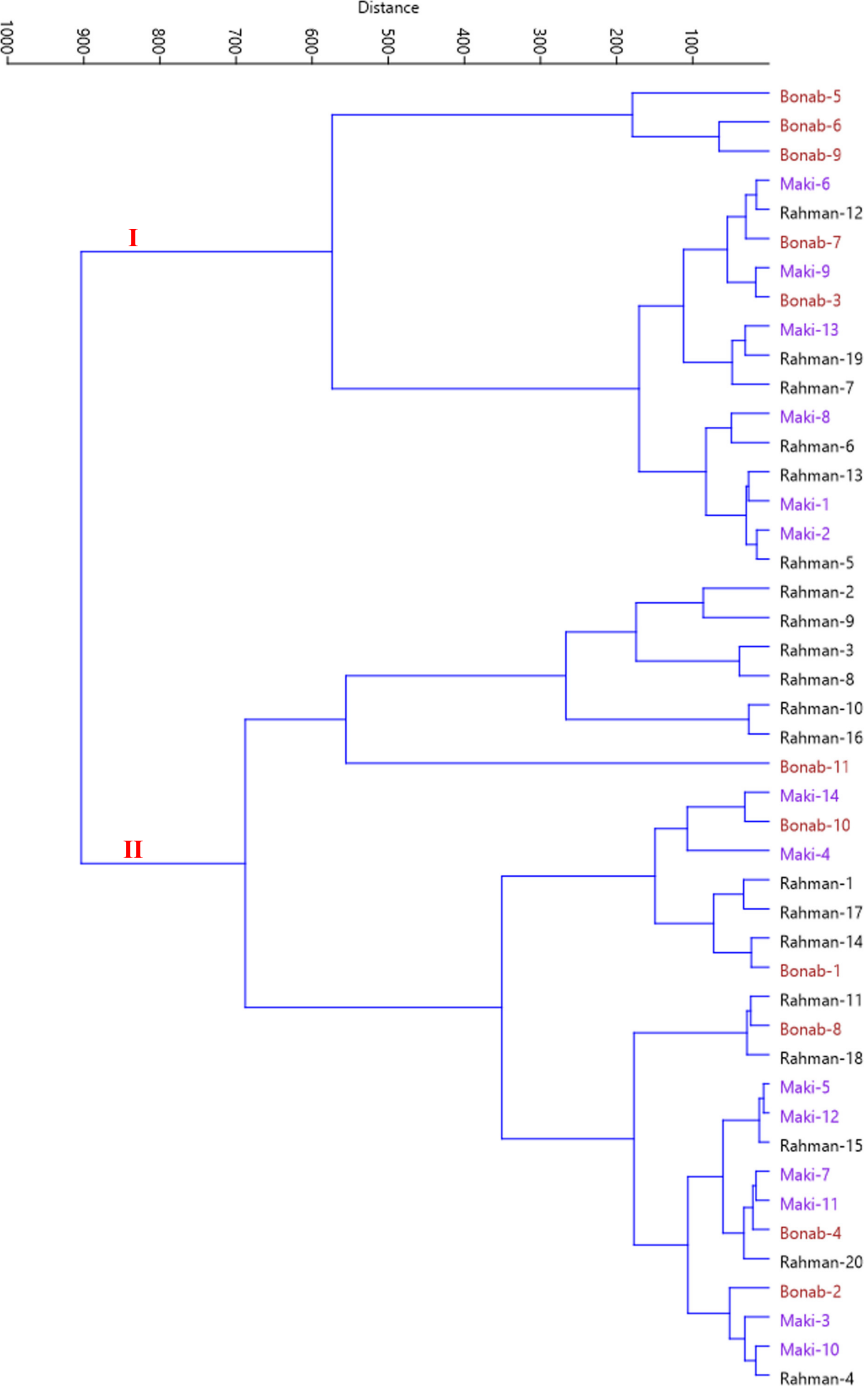
Ward cluster analysis of the studied *M. orientalis* accessions based on morphological traits using Euclidean distances

The scatter plot prepared according to the PC1 and PC2 (25.09% of total variance) reflected the relationship among the accessions in terms of phenotypic characteristics. The plot distributed accessions into four sides and showed significant differences for some traits (Figure 2). Also, the Ward dendrogram reflected the similarities and dissimilarities among the accessions based on the qualitative and quantitative variables measured. The most significant result was represented by the identification of two separate main clusters of the accessions based on morphological characteristics (Figure 3). Cluster I included 17 accessions, while cluster II included 28 accessions forming two subclusters.

The results showed that the studied germplasm have high phenotypic variation. Similarly, high phenotypic variability was reported in *M. orientalis* collections from different countries (Aldwinckle et al., 2002; Ercisli et al., 2004; Hofer et al., 2013; Khadivi et al., 2020). These studies indicated that high diversity in pomological and leaf‐related traits could be used as an efficient marker system to discriminate between the apple accessions.

*M. orientalis* has been reported as one of the possible ancestors of domestic apples. The traits such as late blooming, adaptation to a variety of environmental conditions, long storage life, and disease resistance are found in *M. orientalis*, which can be used in breeding programs to improve domestic apple cultivars (*M. × domestica*). For instance, several wild accessions of *M. orientalis* with resistance to apple scab (*Venturia inaequalis*) and powdery mildew (*Podosphaera leucotricha*) (Ponomarenko, 1992) and also with resistance to fire blight (*Erwinia amylovora*), apple scab, and cedar‐apple rust (*Gymnosporangium juniperi‐virginianae*) have been revealed (Volk et al., 2008, 2009). The current findings support the view that pomological and leaf characteristics are reliable in estimating genetic relationships among apple accessions and can be used efficiently for discrimination. Morphological traits are very helpful in the identification and evaluation of apple germplasm. Phenotypic characterization is always needed and should be included in any program of conservation and use of genetic resources (Aldwinckle et al., 2002; Ercisli et al., 2004; Hofer et al., 2013).

## 4 CONCLUSION

The current plant expedition targeted wild *M. orientalis* with the goal of enhanced germplasm representation in gene banks. A high phenotypic diversity within the collected material of *M. orientalis* was indicated. The diversity existing in the indigenous wild *M. orientalis* could further add new genetic information in the global gene pool of *Malus* species. The present study confirmed the necessity of preserving this unique genetic resource and continuing its study no matter the fact that in practice. The conservation of a wide range of genetic variability and progress in long‐term breeding programs should be the main goal because greater genetic diversity would be desirable for future development of innovative and market‐driven cultivars.

## CONFLICT OF INTEREST

The authors declare no conflict of interest.

## Data Availability

The data that support the findings of this study are available from the corresponding author upon reasonable request.
